# RNA Catabolites Contribute to the Nitrogen Pool and Support Growth Recovery of Wheat

**DOI:** 10.3389/fpls.2018.01539

**Published:** 2018-11-02

**Authors:** Vanessa Jane Melino, Alberto Casartelli, Jessey George, Thusitha Rupasinghe, Ute Roessner, Mamoru Okamoto, Sigrid Heuer

**Affiliations:** ^1^Waite Research Institute, University of Adelaide, Glen Osmond, SA, Australia; ^2^School of Agriculture and Food, University of Melbourne, Parkville, VIC, Australia; ^3^Metabolomics Australia, School of Biosciences, The University of Melbourne, Parkville, VIC, Australia; ^4^Department of Plant Biology and Crop Science, Rothamsted Research, Harpenden, United Kingdom

**Keywords:** nitrogen recycling, nitrate, purines, nucleosides, *Triticum aestivum*, equilibrative nucleoside transporter, ribonuclease, adenosine kinase

## Abstract

Turn-over of RNA and catabolism of nucleotides releases one to four ammonia molecules; the released nutrients being reassimilated into primary metabolism. Preliminary evidence indicates that monocots store high levels of free nucleotides and nucleosides but their potential as a source of internal organic nitrogen for use and remobilization is uncharted. Early tillering wheat plants were therefore starved of N over a 5-day time-course with examination of nucleic acid yields in whole shoots, young and old leaves and roots. Nucleic acids constituted ∼4% of the total N pool of N starved wheat plants, which was comparable with the N available from nitrate (NO_3_^-^) and greater than that available from the sum of 20 proteinogenic amino acids. Methods were optimized to detect nucleotide (purine and pyrimidine) metabolites, and wheat orthologs of RNA degradation (*TaRNS*), nucleoside transport (*TaENT1, TaENT3*) and salvage (*TaADK*) were identified. It was found that N starved wheat roots actively catabolised RNA and specific purines but accumulated pyrimidines. Reduced levels of RNA corresponded with induction of *TaRNS2, TaENT1, TaENT3*, and *TaADK* in the roots. Reduced levels of GMP, guanine, xanthine, allantoin, allantoate and glyoxylate in N starved roots correlated with accumulation of allantoate and glyoxylate in the oldest leaf, suggesting translocation of allantoin. Furthermore, N starved wheat plants exogenously supplied with N in the form of purine catabolites grew and photosynthesized as well as those plants re-supplied with NO_3_^-^. These results support the hypothesis that the nitrogen and carbon recovered from purine metabolism can support wheat growth.

## Introduction

Plant growth and development is reliant on an adequate supply of micro- and macro-nutrients. Nucleotides (purine and pyrimidines), which are composed of the macro-nutrients nitrogen (N), phosphorus (P) and carbon (C), are the building blocks of DNA and RNA. The majority of cellular RNA can be found in complexes with proteins such as ribosomes (Kraft et al., 2008), where they play an important role in protein synthesis. Ribosomal subunits are targets of degradation under nutrient limitation in a process first identified in yeast termed ‘ribophagy’ (Kraft et al., 2008). This pathway is distinct from the ribosome quality control pathways responsible for eliminating defective ribosomes (Liu and Bassham, 2012; Bassham and MacIntosh, 2017). A ribophagy-like pathway was proposed to be conserved in plants involving the autophagy genes *ATG5* and *ATG9* and a type II ribonuclease (RNS2) that resides in the vacuole and the ER (MacIntosh et al., 2010; Floyd et al., 2015). RNS2 defective mutants have longer lived rRNA, with most of the RNA accumulating in vacuoles, providing evidence of its function in the decay of rRNA (Hillwig et al., 2011). It has been proposed that this pathway is not only important in plants for nutrient recycling but also to maintain homeostasis under normal growth conditions as evidenced by induction of constitutive autophagy when this pathway is disrupted (Hillwig et al., 2011). Although *RNS2* is expressed at a high level in all tissues, expression is enhanced further under senescence and during inorganic phosphate starvation (Taylor et al., 1993; Liang et al., 2002).

**FIGURE 1:**
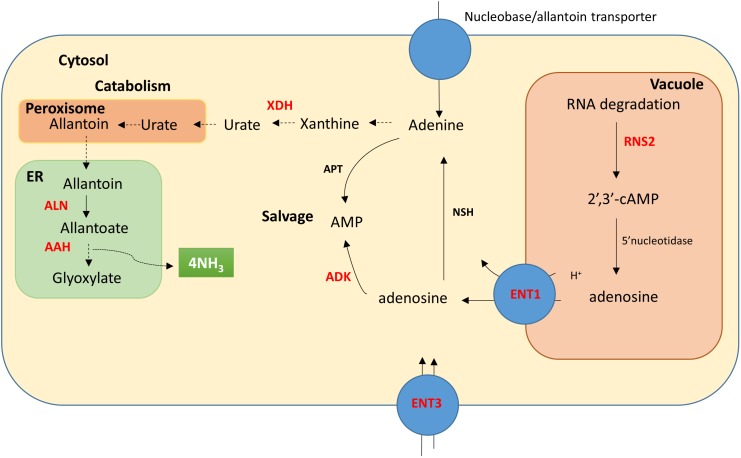
Diagram of the purine pathway characterized in Arabidopsis adapted from Bernard et al. (2011). The diagram shows the cellular localization of RNA degradation, purine salvage and catabolism. Proteins (enzymes and transporters) that were examined here at the gene transcriptional level are shown in red; RNS2, ribonuclease type II; ENT1, equilibrative nucleoside transporter 1; ENT3, equlibrative nucleoside transporter 3; XDH, xanthine dehydrogenase; ADK, adenosine kinase; ALN, allantoinase and AAH, allantoate amidohydrolase. Additional enzymes shown include NSH, nucleoside hydrolase and APT, adenine phosphoribosyltransferase. Cellular localization of these proteins is not known in wheat.

Vacuolar RNA degradation by RNS2 produces 2′nucleoside monophosphates (i.e., 2′,3′-cAMP, Figure 1) which can be dephosphorylated by vacuolar phosphatases or phosphorylases. In Arabidopsis, these nucleosides can then be transported to the cytoplasm by the tonoplast localized equilibrative nucleoside transporter ENT1 (Bernard et al., 2011) (Figure 1). Salvaged nucleosides can then undergo one of three fates; cell-to-cell or long-distance transport, nucleotide synthesis or catabolism (Möhlmann et al., 2010). All Arabidopsis ENT proteins identified (ENT1, 3, 4, 6, and 7) can transport the purine nucleosides adenosine and guanosine and the pyrimidine nucleosides cytidine and uridine (Möhlmann et al., 2001; Li et al., 2003; Wormit et al., 2004; Chen et al., 2006; Traub et al., 2007). AtENT3 was shown to be required for uptake of nucleosides by seedlings as an AtENT3 defective mutant was resistant to the toxic uridine analog fluorouridine (Traub et al., 2007). Furthermore, both expression of *ENT1* and *ENT3* and nucleoside import was increased in Arabidopsis upon N stress (Cornelius et al., 2012).

Nucleotides synthesized from the salvage pathway come at a significantly reduced energetic cost than through *de novo* synthesis (Zrenner et al., 2006; Jung et al., 2009). Nucleosides and nucleobases remobilized from senescing tissue or excess from sources of reserve tissue (i.e., seeds) serve as precursors for the salvage pathway. It has been proposed that the salvage pathway predominately operates in early stages of germination, flowering, pollen tube growth and seed set (Zrenner et al., 2006; Girke et al., 2014). Nucleotides are salvaged from nucleoside monophosphates by the activity of phosphoribosyl transferases or nucleoside kinases. Adenosine kinase (ADK), for example, catalyzes the synthesis of adenine monophosphate from adenosine and adenosine triphosphate (ATP), (Moffatt et al., 2002; Figure 1). The third fate for nucleosides is catabolism where nucleosidases (NSH) remove the ribose sugar from nucleosides to form nucleobases (Zrenner et al., 2006), (Figure 1). Indeed, the activity of an Arabidopsis nucleoside hydrolase (AtNSH1) was shown to be essential to maintain a balance of the nitrogen pools within nucleotides and amino acids (Jung et al., 2009, 2011). Nucleosides and nucleobases can be catabolised via the purine and pyrimidine catabolic pathways liberating four and one molecule of ammonia, respectively (Figure 1) presumably for assimilation by the glutamine synthetase (GS) and glutamate synthase (GOGAT) cycle thereby recycling salvaged nitrogen (Girke et al., 2014).

Preliminary evidence suggests that monocots store high levels of free nucleotides and nucleosides (Sawert et al., 1988). The total nucleotide content of wheat flag leaves was reported to be 2,660 nmol per gram dry weight compared to 400 nmol per gram dry weight in young tobacco leaves (Wagner and Backer, 1992). This is likely to be an underestimate of the actual nucleotide pool given the use of acid hydrolysis steps and less sensitive instruments for metabolite detection. Bread wheat (*Triticum aestivum* L.) is hexaploid with a predicted genome size of 16 Gb (Arumuganathan and Earle, 1991; Appels et al., 2018). Since there are two and five nitrogen elements per pyrimidine and purine nucleotide respectively, each copy of this large genome and the RNA species required for transcription, stores significant amounts of N. It is therefore probable that that under conditions of N stress, wheat can utilize nucleic acids and derivatives as an N (and P) source. This could be for immediate catabolism, allowing release of ammonia for reassimilation, or in the salvage pathway to replenish nucleotide pools that support numerous critical metabolic processes.

In this study, the potential role of nucleic acids as a source of N was examined in two bread wheat genotypes differing in nitrogen use efficiency (Mahjourimajd et al., 2016). Early tillering wheat plants were exposed to mild (24 h), mid (3 days) and severe (5 days) N starvation; the severity of the stress was demonstrated by changes in plant growth and plant N nutritional status. Catabolism of nucleic acids was compared with the induction of key genes involved in RNA degradation and nucleoside transport. A Liquid Chromatography triple quad Mass Spectrometry (LC-QQQ-MS) method was optimized to quantify and authenticate purine and pyrimidine metabolites demonstrating a dynamic interaction of these metabolites and showing preferential degradation of purines in wheat under N stress. The role of this pathway in supporting plant growth was further explored by exogenous supply of purines to N starved wheat plants.

## Materials and Methods

### Plant Growth

South Australian bread wheat (*Triticum aestivum*) lines differing in yields under varying N levels were selected for this experiment. The breeding line RAC875 (RAC655/3/Sr21/4^∗^LANCE//4^∗^BAYONET) is used in Australian breeding programs for its superior drought tolerance (Izanloo et al., 2008). The high yielding commercial cultivar Mace (Wyalkatchem/Stylet) was shown to yield well across different Australian environments (National Variety Trial, 2014). Mace also consistently showed high NUE across four sites where there was a significant genotype by nitrogen interaction (Mahjourimajd et al., 2016). Preliminary evidence suggests greater nitrogen use efficiency (NUE) of Mace (112.6 kg GY kg^-1^ N) as compared to RAC875 (108.1 kg GY kg^-1^ N) in a single site in South Australia, although both genotypes ranked amongst the top 8 (Mahjourimajd et al., 2016).

Seeds were germinated on filter paper moistened with reverse osmosis water for 48 h at 22°C in the dark. Evenly sized imbibed seeds with 1 cm radicles were transferred to separate 120 L ebb and flow hydroponic systems which had a 13-min fill/drain cycle. Plants were grown on mesh collars within tubes (300 mm L × 50 mm W), which kept the roots of adjacent plants separate, but allowed free access to solution. The nutrient solution was a modified Johnson’s solution (Johnson et al., 1957) as detailed in Melino et al. (2015) but with 1 mM N as NO_3_^-^. Nutrient solution was maintained at pH 5.9 and replaced every 7 days. Plants were grown for 14 days (Z21, first tiller emerged) before applying one of two nitrogen treatments; sufficient nitrogen, +N (1 mM N as NO_3_^-^) or N starvation/stress, -N (0 mM N) but with all other nutrients equivalent between the two treatments. The hydroponic system was situated in a controlled environment room with a day: night cycle of 12 h: 12 h, 18°C: 15°C, at a flux density at canopy level of 640 μmol m^-2^s-^1^ (Experiment 2) and 300 μmol m^-2^s-^1^ (Experiments 1 and 3). Plants were grown in equivalent systems, but Experiment 2 was performed at a different time and in a different growth room to Experiments 1 and 3.

#### Experiment 1: Whole Shoot Assessment of N Composition

To quantify the proportion of nitrogen in RNA and DNA as compared to the total nitrogen pool, 14-day old plants (Mace and RAC875) were grown for a further 5 days under either +N (1 mM N as NO_3_^-^) or -N (0 mM N) conditions before harvesting (19-day old plants). Six replicates per genotype per treatment were used. Whole shoot was weighed, and snap-frozen in liquid nitrogen before homogenization with ball bearings at 1000 rpm for 1 min, 5 times, using an automated system (2010 Geno/Grinder^®^) before grinding by hand under liquid nitrogen to a fine powder.

#### Experiment 2: Time Course and Tissue Specific Analyses

Fourteen-day old wheat plants (Mace and RAC875) grown as described in ‘Plant Growth’ were exposed to the treatment and harvested over a time course to more closely assess phenotypic and biochemical changes under N starvation. Plants were harvested at 1 day (15 days old), 3 days (17 days old) or 5 days (19 days old) after application of the nitrogen treatment: +N (1 mM N as NO_3_^-^) or –N (0 mM N). The number of tillers was recorded. Roots and both the youngest fully expanded leaf (YFEL) and the oldest leaf from the main stem were harvested, weighed and snap-frozen. The YFEL was considered fully elongated when the leaf blade folded away from the stem exposing the ligule. To provide sufficient material for the biochemical analyses, three plants were pooled per replicate with six replicates (a total of 18 plants per genotype and per treatment). A complete randomized design of plant genotypes and time points was used for allocation to and positioning within tanks; the technical replicates were arranged in a partial plot design.

#### Experiment 3: Assessing the Contribution of Recycled N to Plant Growth

Mace plants were grown to compare the growth of wheat supplied with organic nitrogen (purine catabolites) or inorganic nitrogen (NO_3_^-^). Fourteen-day old plants were grown for 24 h in conditions of either + N (1 mM N as NO_3_^-^) or –N (0 mM N). After 24 h the -N plants were transferred to 4 L hydroponic units (6 plants each) where N was resupplied either as NO_3_^-^ (rsNO_3_^-^, 1 mM), xanthine (rsXanth, 0.25 mM) or allantoin (rsAlnt, 0.25 mM) or maintained under -N conditions. Nutrient solutions were maintained at pH 5.9 and replaced every 2 days when grown in 4 L hydroponic units. Measurements (photosynthetic rate and chlorophyll) were taken 8 days after resupply and 22 days old plants were harvested for biomass and N analysis.

### Photosynthetic Rate and Chlorophyll Measurements

Photosynthetic rates were measured using a portable LI-6400XT gas analysis system with a fluorescence chamber (Li-Cor, Lincoln, NE, United States) set with the following parameters: 25°C leaf temperature, 300 μmol s^-1^ CO_2_ flow rate to the sample cell, 400 μmol CO_2_ mol^-1^ reference cell CO_2_, 1500 PAR (μmol m^-2^ s^-1^). Measurements were performed on the YFEL of the main stem between 10:00 AM and 02:00 PM. Photosynthetic rate values were calculated with an adjusted area that accounted for leaf width that was estimated from scanned leaf pictures using ImageJ software ^1^. Chlorophyll content was measured using a portable chlorophyll meter (SPAD-502, Konica Minolta, Tokyo, Japan). Measurements were taken by averaging reads at the bottom, the center and the tip of the YFEL.

### DNA Extraction and Quantification

Shoot (50 mg) or root tissue (70 mg) was mixed with 600 μl of phenol/chloroform/iso-amyl alcohol (25:24:1, PCI) by rotation for 20 min. Samples were centrifuged and the supernatant was collected. 600 μl PCI was added to the pellet and the mixing and centrifugation steps were repeated. Ethanol precipitation [60 μl 3M sodium acetate, pH 4.8 and 600 μl 100% (v/v) isopropanol], a 20-min incubation and centrifugation steps were used to concentrate and pellet the DNA. DNA pellets were washed in 1 ml of 75% (v/v) ethanol before removing the ethanol and final resuspension in 50 μl of Ribonuclease A (RNase A, Sigma–Aldrich) prepared in TE buffer (10 mM Tris-HCl and 1 mM disodium EDTA, pH 8.0) to remove RNA. DNA samples were incubated at 60°C for 10 min. DNA quality was assessed by gel electrophoresis. Diluted samples were aliquoted into a black-walled, clear-bottom 96-well microplate (Molecular Probes^®^, Thermo Fisher Scientific, United States). DNA was quantified using the Quant-iT dsDNA broad range kit (Life Technologies, United States) according to manufacturers’ instructions. Only double stranded DNA molecules are detected using this method. Fluorescent signals were measured using a microplate reader (Optima, BMG LABTECH) with Excitation/Emission maxima of 485/520 nm.

### RNA Extraction and Quantification

Fifty mg of homogenized shoot tissue or 100 mg of homogenized root tissue was extracted in 1ml of TRIzol Reagent (Thermo Fisher Scientific, United States) according to manufacturers’ instructions. After air-drying the RNA, it was resuspended in 40 μl of RNase-free water (Life Technologies) and incubated at 60°C for 15 min. RNA was quantified using the Quant-iT RNA Assay Kit (Life Technologies). This kit is intended for quantification of total RNA, rRNA and mRNA > 20 nucleotides in length. Ten μl of diluted RNA within the range of 5–100 ng in 200 μl final volume was prepared according to manufacturers’ instructions and aliquoted in to a black-walled, clear-bottom 96-well microplate (Molecular Probes^®^, Thermo Fisher Scientific, United States). Fluorescent signals were measured using a microplate reader (Optima, BMG LABTECH) with Excitation/Emission maxima of 640/690 nm.

### RNA Quality Assessment

RNA quality was assessed by microfluidic gel electrophoresis using an RNA 6000 Nano Kit (Agilent, United States) and an Agilent 2100 Bioanalyzer and performed at the David Gunn Genomics Facility (SAHMRI, Adelaide). The 25S to 18S ratio was determined to range from 1.16 to 1.24 which is within the theoretically desired range.

### Identification of Wheat Genes

The genes were identified by BLASTP search using *Arabidopsis thaliana* protein sequences derived from NCBI^2^ as a query in Phytozome^3^. *Brachypodium distachyon* protein sequences were then used for a TBLASTN search against the Chinese Spring TGACv1 genome assembly^4^ (Clavijo et al., 2017). The copy number has been confirmed in the latest release (the IWSGC RefSeq v 1.0 Assembly) in EnsemblePlants. The wheat allantoinase (*TaALN*) and allantoate amidohydrolase (*TaAAH*) genes were identified by Casartelli et al. (Under Review). Amino acid sequences from different plant species were compared using Geneious 11.0.4 with standard alignment settings.

### Amplification of Target Genes

RNA quality was initially assessed on a 1% (w/v) agarose gel by electrophoresis (80 V for 25 min) after incubating with RNA loading dye (NEB) at 65°C for 10 min. Both the intactness of the RNA and the absence of gDNA from the RNA samples was confirmed by cDNA synthesis using SuperScript III (Life Technologies) according to manufacturers’ instructions and PCR amplification using Standard Taq Polymerase and reagents (NEB) according to manufacturers’ instructions. Primers targeting a fragment of wheat genes (Supplementary Table 1) but that overlap an intron were used to distinguish cDNA from gDNA; DNA extracted as described above was used as a positive control.

### qRT-PCR

One μg of total RNA was used for cDNA synthesis using the SuperScript^®^ III kit (Thermo Fisher Scientific) as per manufacturers’ instructions. Quantitative real–time PCR was performed with KAPA SYBR^®^ Fast qPCR kit Master Mix, and amplification was real–time monitored on a QuantStudio 6 Flex Real-Time PCR System (Applied Biosystems). Gene-specific primers targeted to amplify all three homeologs simultaneously were designed (Supplementary Table 1) and the specificity of each pair was verified by melt curve analysis and sequencing of the products. Change in gene expression (calibrated normalized relative quantities, CNRQ) were calculated using qBASE+ software (Hellemans et al., 2007). Specifically, gene expression was normalized to wheat reference genes (*cyclophilin* and *elongation factor alpha*) and then scaled to the 1 day +N weighted mean.

(1) $$\begin{array}{cc} NRQ=\frac{E_{goi}^{\Delta Ct,goi}}{f\sqrt{\prod_{o}^{f}E_{ref_{o}}^{\Delta Ct,ref_{o}}}} & \begin{array}{c} E\;:\;\mathrm{efficiency}\\ \Delta Ct\;:\;\mathrm{delta}-\mathrm{Ct}\\ \mathrm{Ct}\;:\;\mathrm{cycle}\;\mathrm{threshold}\\ \mathrm{goi}\;:\;\mathrm{gene}\;\mathrm{of}\;\mathrm{interest}\\ \mathrm{ref}\;:\;\mathrm{reference} \end{array} \end{array}$$

### Total Nitrogen

For experiment 1, total N was determined using a PDZ Europa ANCA-GSL elemental analyzer interfaced to a PDZ Europa 20–20 isotope ratio mass spectrometer (Sercon Ltd, Cheshire, United Kingdom) performed at UC Davies Stable Isotope Facility^5^.

For experiment 3, 75–100 mg DW of homogenized leaf samples was used for the analysis with a rapid N exceed (Elementar, Germany). Aspartic acid (250 mg) was used as standard for calibration.

### Nitrate Quantification

Nitrate content in plant extracts was determined by nitration of salicylic acid under highly acidic conditions and absorbance of the chromophore at 410 nm in a basic solution according to Cataldo et al. (1975) with modifications as described. Homogenized tissue (20 mg FW) was boiled in 1 ml of MQ water for 20 min. Samples were cooled on ice and centrifuged at 16,000 rpm for 15 min. The supernatant or NO_3_^-^ standard was then decanted into a 1.5 ml tube and mixed with 200 μl of 5% (w/v) salicylic acid concentrated sulphuric acid and transferred into a 10 ml tube containing 4.75 ml of 2N sodium hydroxide. Samples were mixed and incubated at room temperature for 20 min. Two hundred μl of each reaction mix was assessed in a flat-bottom, transparent, 96-well microplate. Absorbance was read at 410 nm in a microplate reader (Optima, BMG LABTECH).

### Amino Acid and Ammonium Analysis

Free amino acids and ammonium were extracted from 20 mg DW (freeze dried, experiment 2) or 30 mg FW (snap-frozen, experiment 1) with 0.5 ml of 10 mM sodium acetate containing 0.25 mM Norvaline (internal standard). Amino acids and ammonium were detected on a Waters AcquityTM UPLC system using the Waters AccQ-Tag Ultra Chemistry Kit following the manufacturers’ instructions (Waters Corp., United States). Chromatograms were analyzed with Empower 3^®^ Software (Waters Corp., United States) and amino acids were authenticated and quantified using standards (Waters Corp. and Sigma–Aldrich).

### Nitrogen Pool Calculations

The N content of DNA was calculated by multiplying the DNA yield (g.g FW^-1^) by 39.53%; which is the calculated percentage of the genome composed of nitrogen based on the assumptions of a 50% GC content, a 16 Giga bp genome size and with known molecular weights of each nucleobase. The nitrogen content of RNA was determined by multiplying the RNA yield (g.g FW^-1^) by 14.6%; which is the calculated percentage of total rRNA composed of nitrogen based on the assumption of a 50% GC content and known molecular weights and lengths of tobacco leaf rRNA species. For simplicity, other RNA species were not considered in these calculations. RNA of these samples was assessed using microfluidic gel electrophoresis to determine the area of each rRNA species as a percentage of the total rRNA area as shown here: 25S (37.45%), 18S (30.71%), 23S (14.78%), 16S (10.5%), and 5S (6.56%). The nitrogen content of ammonium and NO_3_^-^ was calculated based on the known molecular weight of nitrogen and NO_3_^-^. The nitrogen content of the sum of 20 basic amino acids is presented here; calculations were based on the known molecular weight of each individual amino acids prior to their addition together.

### Detection and Quantification of Metabolites in the Purine and Pyrimidine Pathways

Ten mg of sample (freeze dried, DW) was weighed and metabolites extracted in 500 μl of water containing 25 μM of the internal standard (DL-Allantoin-5-^13^C,1-^15^N, 98 atom %, Sigma–Aldrich). Spiked samples were sonicated for 30 min at room temperature then centrifuged for 15 min at 15,000 *g* at 4°C. Samples were stored at -20°C until analysis. Nucleotides, nucleosides, nucleobases and downstream catabolites were separated using ultra-high performance liquid chromatography coupled to an triple quadrupole mass spectrometry (QQQ6490-MS, Agilent, Singapore) according to the method of Guo et al. (2013) with some modifications. An ACQUITY UPLC BEH Amide column (2.1 mm × 100 mm, 1.7 μm), (Waters, NSW, Australia) was used for compound separation. The mobile phase composition included A: 0.8% acetic acid and 10 mM ammonium acetate in water and B: 0.1% acetic acid in acetonitrile with a gradient elution: 0–6 min. 10% A; 6–9 min, 10–40% A; 9–12 min; 40–50% A; 12–18 min; 10% A to equilibrate the column to initial conditions. The flow rate of the mobile phase was maintained at 0.4 ml.min^-1^ and the column temperature was maintained at 35°C. The needle wash was 20% (v/v) acetonitrile in water with sample injection volume of 1 μl. The separated compounds were detected with an Agilent QQQ6490 mass spectrometer and analysis was operated by Agilent MassHunter acquisition software, version 7. All the compounds were quantified based on calibration curves prepared using authentic standards (Sigma–Aldrich).

Mass spectrometry detection was performed using ESI source operated in positive ion mode. The source parameters were set as; capillary voltage 3.5 kV, iFunnel high pressure RF in positive and negative mode at 130 V, low pressure RF in positive and negative mode at 60 V; source temperature 250°C, sheath gas temperature 400C°, gas flow 12 l/min, sheath gas flow 12 l.min^-1^, fragmentor voltage 380 V and cell accelerator 5 V. Data was collected using multiple reaction monitoring (MRM) with similar collision energy as published by Guo et al. (2013). Dwell time for each compound was set as 10 ms and data was quantified using MassHunter Quant software version 7.

Peak area was normalized to the internal standard (norvaline) and divided by the dry weight of the samples. Metabolites in glyoxylate/dicarboxylate metabolism including citric acid, glyoxylic acid and succinic acid were authenticated. Purine metabolites adenine, adenosine, allantoate, allantoin, 2′-deoxyguanosine, guanine, hypoxanthine, guanine monophosphate (GMP), glyoxylate, urate and xanthine were authenticated. Pyrimidine metabolites cytidine, cytosine, 2′-deoxycytidine, 3′-deoxyuridine and uridine were authenticated. Remaining purine and pyrimidine metabolites could not be detected using this method. Additionally, allantoin could not be detected in the oldest leaf and in only some of the YFEL replicates, however, it was detected in the roots. The most likely explanation is that allantoin levels in these leaf samples was at the limit of detection; LOD of 2.5 μM.

### Statistical Analysis

All biochemical and metabolic data was analyzed using GenStat 12th Edition. Data was assessed for homogeneity of variance and for normality of the residuals. Skewed data was transformed. Outliers were detected as large residuals compared to the standard error of the units and true outliers were set to missing values. ANOVA was performed to determine the factorial interactions. Multiple comparisons were performed on significant interactions using the Bonferroni *post hoc* tests with a significance level of 0.05. The response to N starvation was calculated using log2(treatment/reference) where the treatment was the mean of the biological replicates (*n* = 4–6) grown under N starvation (-N) and the reference was the mean of the biological replicates (*n* = 4–6) grown under sufficient N (+N). Comparisons between two means for the transcriptional data was performed using a *T*-test and *post hoc* analysis using the Holm–Sidak method with significance reported as ^∗∗∗^*p* ≤ 0.001, ^∗∗^*p* ≤ 0.02, ^∗^*p* ≤ 0.05.

## Results

### Changes in Nitrogen Composition Under N Starvation Differ Between Genotypes

To demonstrate the N status of the wheat plants and the severity of the N stress imposed, shoot fresh weight (FW), total N, NO_3_^-^, ammonium and the sum of 20 proteinogenic amino acids (total amino acids) were quantified in wheat plants grown under N sufficiency (+N) and N starvation/stress (-N). The results show that although the shoot fresh weight of RAC875 was greater than Mace (Supplementary Table 2, *p* < 0.001), the total shoot nitrogen content on a per gram basis was equivalent between Mace and RAC875 (Supplementary Table 2). Plants grown under severe N stress were smaller (reduced fresh weight) and accumulated approximately half the total nitrogen content of the +N control plants (Supplementary Table 2, *p* < 0.001). The shoot NO_3_^-^ content was equivalent between Mace and RAC875 grown under +N conditions but levels were reduced by 4.5 to 5.9-fold in plants grown under -N conditions (Supplementary Table 2, *p* < 0.001). Shoot ammonium levels were equivalent between genotypes and treatments (Supplementary Table 2). In contrast, RAC875 accumulated higher total amino acids than Mace under +N conditions and levels were reduced in both genotypes under -N conditions (Supplementary Table 2, *p* < 0.001).

**FIGURE 2:**
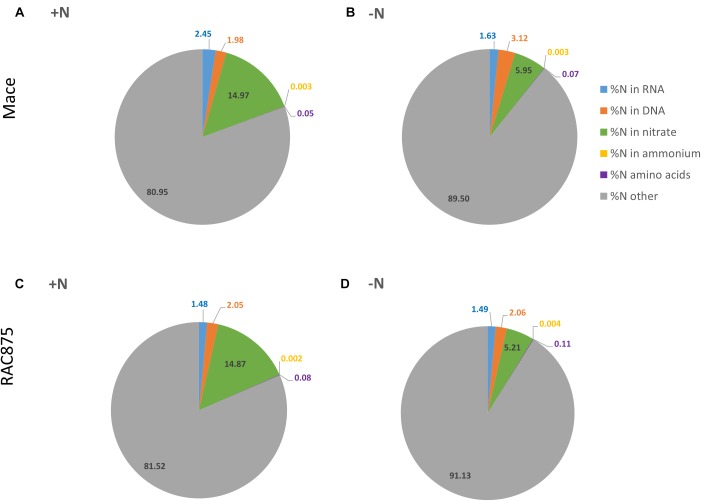
Nitrogen sources in wheat shoots as a percentage of total nitrogen. Plants of the genotypes Mace **(A,B)** and RAC875 **(C,D)** were grown for 14 days with full nutrition before applying the treatments +N (1 mM N; **A,C**) or –N (0 mM N; **B,D**) for 5 days (*Experiment 1*). Experimental values were determined for DNA, RNA, nitrate (NO_3_^-^), ammonium, amino acids (20 total basic amino acids) prior to calculations of their nitrogen (N) content. The N content of DNA and RNA was calculated by multiplying the yield (g. gFW^-1^) by 39.53 and 14.6% respectively. The N content of ammonium and NO_3_^-^ was calculated based on the known molecular weight of nitrogen and NO_3_^-^. The N content of the sum of 20 basic amino acids is presented here; calculations were based on the known molecular weight of each individual amino acids prior to their addition together.

The nitrogen content of ammonium, amino acids and NO_3_^-^ was calculated as a percentage of the total N pool to determine their relative contribution under both +N and -N growth conditions. Ammonium accounted for on average 0.003% of the total N pool; there were no significant differences between genotypes or treatments for %N in ammonium (Figures 2A–D). The N from the free amino acids accounted for 0.05 and 0.08% of the total N pool in +N grown Mace and RAC875 respectively (Figures 2A,C). However, under -N conditions, amino acids accounted for more of the total N pool (0.11%) in RAC875 than under +N conditions (Figure 2D, *p* < 0.05). Nitrate accounted for much more of the total N pool than either ammonium or the total amino acids. Nitrate accounted for approximately 15% of the total N pool under +N conditions and was reduced to 5.2–6.0% of the total N pool under N stress (Figures 2A–D, p < 0.05). Reduction in NO_3_^-^ levels lead to an increased proportion of the total N pool accounted for by ‘other’ (Figures 2A–D, *p* < 0.05). The majority of the ‘other’ proportion of nitrogen was not determined in this study due to the understanding that other soluble and insoluble N sources exist within a plant including nucleotide sugars, amides, amines, polyamines, peptides and proteins.

### N Starved Wheat Plants Have Reduced RNA Yields Which Are Unchanged Relative to the Total N Pool

To determine the relative significance of nucleic acids as a source of N for wheat growing under N limited conditions, RNA and DNA levels were quantified using sensitive and accurate methods and the proportion of N in these nucleic acids calculated using the assumptions described in the M&M. There were no significant genotypic differences in RNA or DNA as a percentage of total N. The results averaged across genotypes grown under N sufficient conditions show that both RNA and DNA accounted for 2.0% of the total N pool of shoots (Figures 2A,C). The proportion of RNA as a percentage of total N under N sufficient conditions (Figures 2A,C) was maintained under N starvation (Figures 2B,D, *p* > 0.1), which suggests that RNA yields reduced by a similar magnitude to that of total N. The proportion of N in DNA as % of total N was statistically unchanged by the treatment in both Mace and RAC875, although there was a non-significant trend of increased N from DNA as % of total N pool in N starved Mace plants, which is clearer when comparing absolute values as below.

**FIGURE 3:**
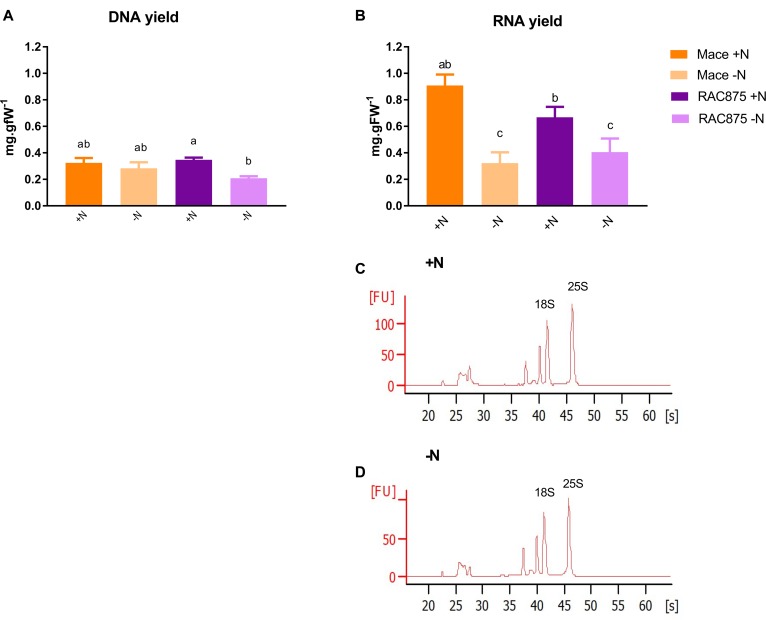
The quantity and quality of nucleic acids of wheat shoots. Total DNA **(A)** and RNA **(B)** yields extracted from Mace and RAC875 whole shoots (*Experiment 1*) as determined by fluorescence assays (Quant-it dsDNA broad range kit and Quant-iT RNA Assay Kit, Life Technologies, United States). **(C,D)** RNA quality assessment via RNA smear analysis using an RNA 6000 Nano Kit (Agilent, United States) and the Agilent 2100 Bioanalyzer. The chromatograms **(C,D)** show the fluorescence (*y*-axis fluorescent units, FU) of rRNA species over time (*x*-axis, seconds, s) with the faster migrating 25S peak at 45.8 s and the slower 18S rRNA peak at 41.35 s. Representative image of a +N treated RAC875 shoot sample **(C)** and a -N treated RAC875 shoot sample **(D)**.

The DNA and RNA yields of Mace and RAC875 were equivalent under N sufficient conditions (Figures 3A,B). The DNA yield of Mace shoots was equivalent between +N and -N treatments but the DNA yield of RAC875 shoots was reduced when grown under N starvation as compared to its respective control (Figure 3A). The RNA yield was reduced in both genotypes under N starvation to an equivalent final RNA yield (Figure 3B). Quality analysis of the RNA using microfluidic gel electrophoresis (Figures 3C,D) shows that although the fluorescence units were reduced in the RNA extracted from -N treated shoots, the RNA was intact as indicated by maintenance of the relative proportion of each ribosomal RNA species. The 25S to 18S ratio was unchanged by the treatment (mean 25S:18S ratio ranged from 1.21 ± 0.02 at -N where *n* = 6 to 1.23 ± 0.02 at +N where *n* = 6; data not shown).

### Shoot Growth but Not Root Growth Is Inhibited Under N Starvation

**FIGURE 4:**
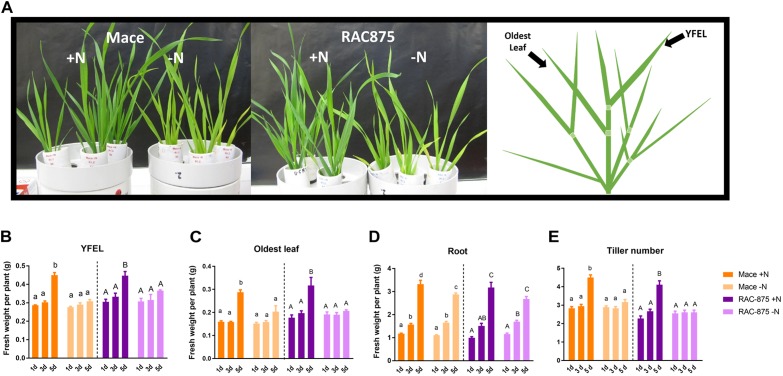
The phenotypic response of wheat genotypes to N starvation. Plants of the genotypes Mace and RAC875 were grown for 14 days with full nutrition before applying the treatments +N (1 mM N) or –N (0 mM N) for 1 day, 3 days or 5 days. *n* = 6 with bulking of 3 plants per n (*Experiment 2*). **(A)** Left to right; Photographs of Mace and RAC875 19-day old wheat plants exposed to either +N or –N conditions for 5 days and illustration of leaf positioning. Fresh weight of the youngest fully expanded leaf (YFEL) **(B)**, oldest leaf **(C)** or roots **(D)**. **(E)** Number of tillers including the main stem. Different letters of the same case indicate significant differences between treatments and time points within a single genotype as determined using a two-way ANOVA with Bonferroni *post hoc* test at significance of *p* < 0.05.

Wheat seedlings of the analyzed two genotypes contrasting in NUE (Mahjourimajd et al., 2016) were grown in full nutrition for 14 days before transfer to either nutrient solution without nitrogen (-N, nitrogen starvation) or nutrient solution with nitrogen (+N, control) and harvested over a time course (mild, 24 h; mid, 3 days or severe, 5 days). The YFEL and the oldest leaf harvested from the main stem (Tiller 0) and the roots provided material to assess whether tissues of different ages regulate nucleotide metabolism differently. Mace and RAC875 plants showed visible signs of accelerated chlorosis of the older leaves only after 5 days of N starvation (Figure 4A), and there were no genotypic differences in terms of measured components (Figures 4B–E) so statistical comparisons shown are between treatments within a single genotype. Five days of N starvation resulted in smaller plants in terms of reduced fresh weight of their YFEL, oldest leaf and a reduced number of tillers (*P* < 0.05, Figures 4B,C,E), however, the roots continued to grow (Figure 4D, *p* < 0.05).

### Nitrate Levels Are Reduced, and the Root Nitrate Transporter Is Induced After 24 h of N Starvation

**FIGURE 5:**
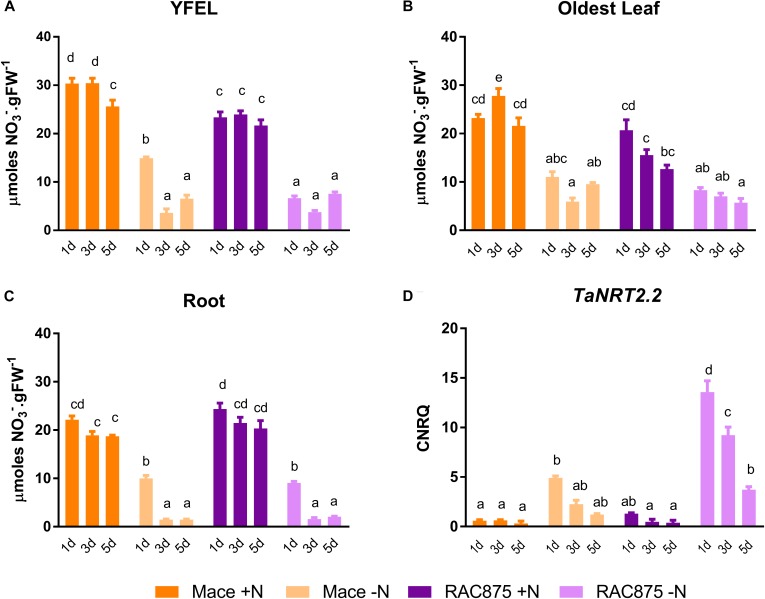
The effects of N starvation on the tissue accumulation of nitrate and the transcription of a root specific nitrate transporter. Nitrate concentration in the **(A)** YFEL, youngest fully expanded leaf, **(B)** oldest leaf on the main stem and the **(C)** root of 14 days old plants of genotypes Mace and RAC875 (*n* = 6 with bulking of 3 plants per n) grown for a further 1 day, 3 days or 5 days exposure to either +N (1 mM N) or –N (0 mM N) growth conditions. **(D)** Transcriptional profile of the NO_3_^-^ transporter (*TaNRT2.2*) in Mace or RAC875 roots only expressed as calibrated normalized relative quantities (CNRQ). Different letters indicate significant differences between treatments, time points and genotypes as determined using a two-way ANOVA with Bonferroni *post hoc* test at significance of *p* < 0.05.

It is now well established that NO_3_^-^ is involved in signaling pathways adjusting plant growth development in response to nutrient availability as reviewed recently by Krouk (2017), it was pertinent to measure NO_3_^-^ levels not only at the whole shoot level but also in specific tissues (Figure 5) where growth was altered in response to N availability. Nitrate yields were higher in the YFEL and the oldest leaf of Mace plants grown under N sufficient conditions than in the same tissue of RAC875 (24 h and 3 days only Figure 5A; 3 days only Figure 5B, *p* < 0.05) whilst the roots of both genotypes accumulated equivalent levels of NO_3_^-^ under both +N and -N growth conditions (Figure 5C). The NO_3_^-^ content in each tissue of both Mace and RAC875 was reduced when plants were grown under mild N starvation as compared to the +N control (Figures 5A–C, *p* < 0.05). Transcription of the gene putatively encoding the root specific NO_3_^-^ transporter *TaNRT2.2* was previously shown to be regulated by plant N status (Melino et al., 2015). This gene was therefore a good candidate to in-directly assess root NO_3_^-^ uptake in response to N availability. *TaNRT2.2* was induced under mild N stress in both Mace and RAC875 with a rapidly declining level of transcription in RAC875 (Figure 5D, *p* < 0.05), indicating that the sensing of N limitations in the shoot and signaling to the root is rapid.

Other important and readily available sources of plant nitrogen such as ammonium and total amino acid content were also assessed here to better determine the N status of these tissues. Root ammonium content was higher than YFEL ammonium content in +N plants (Supplementary Figure 1, *p* < 0.05) whilst ammonium levels were equivalent between tissues of -N plants (Supplementary Figure 1). Ammonium levels were reduced in all tissues of RAC875 and a similar trend, although not always significant was also observed for Mace (Supplementary Figure 1). Total amino acid content was reduced under N starvation only in the oldest leaf of both genotypes and in the roots of RAC875 (Supplementary Figure 1). Although, it was not significant, there was a clear trend of decreasing amino acid levels in N starved Mace roots (Supplementary Figure 1).

### RNA Yields Are Reduced in Young Leaves and Roots Under N Starvation

**FIGURE 6:**
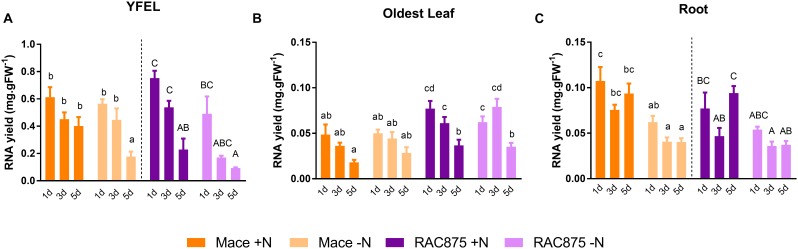
The effects of N starvation on tissue specific RNA yields. Plants of the genotypes Mace and RAC875 were grown for 14 days with full nutrition before applying the treatments +N (1 mM N) or –N (0 mM N) for 1 day, 3 days or 5 days. *n* = 6 with bulking of 3 plants per n. Total RNA **(A–C)** yields extracted from Mace and RAC875 tissues were determined by fluorescence assays (Quant-it dsDNA broad range kit and Quant-iT RNA Assay Kit, Life Technologies, United States). Total RNA yields from YFEL **(A)**, the oldest leaf from the main stem **(B)** and roots **(C)**. The *Y*-axis scaling is equivalent within a tissue. Letters of the same case indicate significant differences between treatments and time points within a single genotype as determined using a two-way ANOVA with Bonferroni *post hoc* test at significance of *p* < 0.05.

A second growth experiment was undertaken over a time course to more closely assess phenotypic and biochemical changes under N starvation. RNA yields of 14-day old plants grown under control conditions for 24 h were 10.5-fold higher in the YFEL (mean 0.626 mg RNA. g FW^-1^) than the oldest leaf (mean 0.060 mg RNA. g FW^-1^) and 8.6-fold higher than the root (mean 0.073 mg RNA. g FW^-1^), (Figures 6A–C, *p* < 0.001). RNA yields were higher in the oldest leaf of RAC875 than the same tissue of Mace in both treatments (Figure 6B), however, genotypic differences were not observed for RNA yields in the YFEL and the root, and therefore statistical comparisons between treatment and time-point means within a single genotype are presented (Figures 6A,C). RNA yields in the YFEL of Mace plants growing under severe N stress were reduced as compared to the control (Figure 6A, *p* < 0.05) but reduced yields were not evident in RAC875 (Figure 6A). Instead RNA yields in RAC875 were steadily decreasing over developmental time in both the control and N starved plants (Figure 6A, 24 h to 5 days, *p* < 0.05). There was no significant treatment effect on RNA yields in the oldest leaf of either genotype (Figure 6B). RNA yields were reduced in Mace roots exposed to N stress (all time-points as compared to the control), but a reduction was only observed in RAC875 roots grown under severe N stress as compared to the control (Figure 6C).

### Genes Putatively Encoding RNA Degradation and Nucleoside Transport Are Up-Regulated in Response to N Starvation

To further characterize tissue specific differences in RNA degradation and nucleoside transport, three genes were identified in bread wheat based on homology to Arabidopsis, rice, maize and Brachypodium as putatively encoding ribonuclease type II (*TaRNS2*), equilibrative nucleoside transporter 1 (*TaENT1*), equilibrative nucleoside transporter 3 (*TaENT3*) and adenosine kinase (*TaADK*). Three homeologs of each gene were identified in bread wheat (Supplementary Table 3). *RNS2* is one of five RNase T2 genes in Arabidopsis and one of eight RNase T2 genes in rice (MacIntosh et al., 2010). Similar to Arabidopsis and rice, a single *RNS2* gene was identified in the wheat genome (*TaRNS2*). TaRNS2 homeologs shares 72 to 74% amino acid identity with the rice *OsRNS2* (Supplementary Table 4A). Although seven paralogs of ENT exist in the Arabidopsis genome (Li and Wang, 2000; Möhlmann et al., 2001; Li et al., 2003; Wormit et al., 2004) and four paralogs in the rice genome (Hirose et al., 2005), only two putative ENT paralogs, *TaENT1* and *TaENT3*, were identified here in the wheat genome (Supplementary Table 3). TaENT1 homeologs share 82% amino acid identity with OsENT1 (Supplementary Table 4B) and TaENT3 homeologs share 82% amino acid identity with OsENT3 (Supplementary Table 4C). Two paralogs of ADK were identified in the Arabidopsis genome (Moffatt et al., 2000) and although activity has been demonstrated in wheat germ (Chen and Eckert, 1977), it was not previously identified at the gene level. Three homeologs of a single copy of TaADK was identified here sharing 83% amino acid identity with AtADK1 (AT3g09820) and 83% amino acid identity with AtADK2 (AT5g03300), (Supplementary Table 4D). The TaADK homeologs also share very high amino acid identity (93%) with the rice ortholog OsADK (Supplementary Table 4D).

**FIGURE 7:**
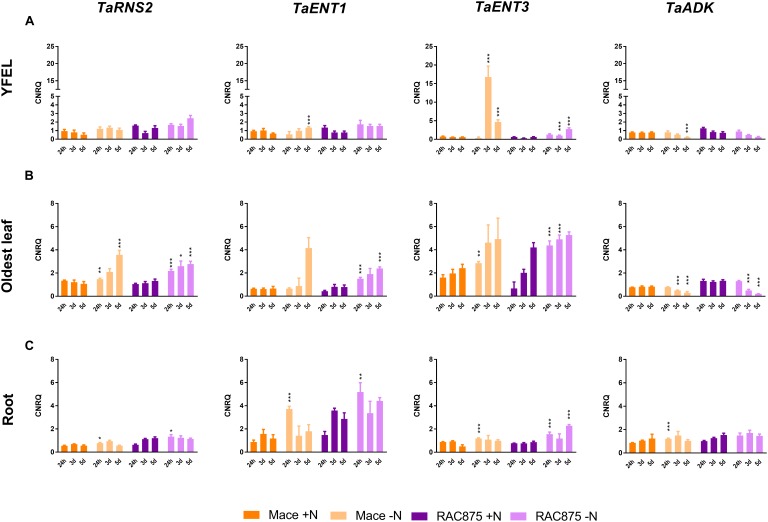
Transcriptional response to N starvation of genes with putative functions in RNA degradation, nucleoside transport and salvage. Plants of the genotypes Mace and RAC875 were grown for 14 days with full nutrition before applying the treatments +N (1 mM N) or –N (0 mM N) for 1 day, 3 days or 5 days. *n* = 6 with bulking of 3 plants per n. Transcriptional profile of the putative ribonuclease type II gene (*TaRNS2*), the putative equilibrative nucleoside transporter 1 gene (*TaENT1*), the putative equilibrative nucleoside transporter 3 gene (*TaENT3*) and the putative adenosine kinase gene (*TaADK*) from Mace and RAC875 youngest fully expanded leaf (YFEL, **A**), oldest leaf **(B)** and roots **(C)** expressed as calibrated normalized relative quantities (CNRQ). Statistical significance between treatments within a single time point and a single genotype at ^∗∗∗^*p* ≤ 0.001, ^∗∗^*p* ≤ 0.02, ^∗^*p* ≤ 0.05 as determined by *T*-test analysis and *post hoc* analysis using the Holm-Sidak method.

Primers for transcriptional analysis presented here were designed to amplify all three homeologs. Transcription of *TaRNS2* was constitutive in the YFEL across time and treatments (Figure 7A). Transcriptional levels of *TaENT1* in the YFEL of Mace increased 2-fold but only when grown under severe N stress as compared to the 5 days + N control (Figure 7A, *p* < 0.05); *TaENT1* levels were unchanged in RAC875. *TaENT3* expression was significantly enhanced in both Mace (28-fold) and RAC875 (3.5-fold) plants grown under mid N stress as compared to their respective controls (Figure 7A, *p* < 0.05). Transcriptional levels of *TaENT3* in the YFEL of Mace plants grown under mid N stress were 17-fold higher than that of RAC875 plants grown under equivalent conditions (Figure 7A, *p* < 0.05). Since levels of *TaENT3* expression in Mace were 17-fold higher than *TaENT1* in the same tissue (Figure 7A, *p* < 0.05), this could indicate preferential reliance on imported nucleosides in the YFEL of Mace rather than degrading and exporting nucleosides from the vacuole locally. This is supported by the lack of *TaRNS2* response to N starvation in the YFEL of either genotype as already mentioned above (Figure 7A).

In contrast to the YFEL, transcriptional levels of *TaRNS2* increased by 1- to 3-fold under N starvation in both the oldest leaf and roots of Mace and RAC875 under N stress as compared to the relevant controls (Figures 7B,C, *p* < 0.05). Transcription of *TaENT1* was also increased in the oldest leaf and roots of RAC875 grown under mild N stress as compared to the 24 h +N control (Figures 7B,C, *p* < 0.05). Transcription of *TaENT1* was 4-fold greater in Mace roots grown under mild N stress than the control (Figure 7C, *p* < 0.05) and there was a non-significant trend of increased transcription when grown under severe N stress in the oldest leaf (Figure 7B). Like-wise transcriptional levels of *TaENT3* were enhanced in the oldest leaf (up to 6.6-fold) and roots (up to 1.8-fold) of RAC875 plants grown under mild N stress (Figures 7B,C, *p* < 0.05). Up-regulation of these genes in the oldest leaf is not surprising since the Arabidopsis paralogs of *RNS2, ENT1* and *ENT3* are all known to be transcriptionally induced by senescence, and the nitrogen stress imposed here accelerated senescence of the older leaves. The putative wheat ortholog of adenosine kinase, *TaADK*, was reduced by 3.5-fold in the YFEL of Mace plants grown under severe N stress (Figure 7A, *p* < 0.05) and with a similar but non-significant trend for the YFEL of N starved RAC875 plants (Figure 7A). *TaADK* levels were also slightly reduced in the oldest leaf of both genotypes grown under mid N stress (Figure 7B, *p* < 0.05). *TaADK* was instead induced (1.4-fold) in the root of Mace plants exposed to mild N stress (Figure 7C, *p* < 0.05) with a similar non-significant trend in RAC875 (Figure 7C).

### Purine Metabolism Differs Between Roots and Leaves Exposed to N Starvation

**FIGURE 8:**
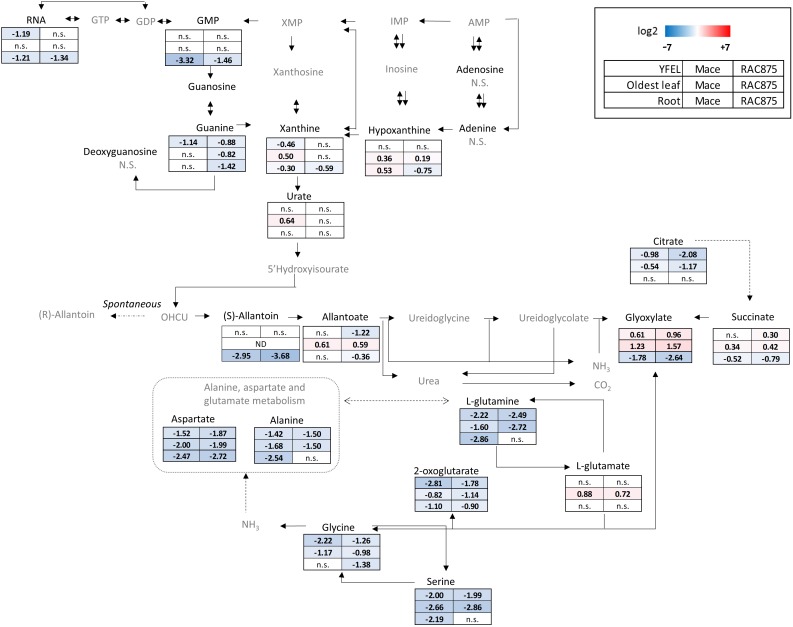
Metabolic map of purine and glyoxylate metabolism in bread wheat in response to N starvation. Bread wheat genotypes Mace and RAC875 were grown for 14 days under N sufficient conditions (+N) before transfer to N starvation conditions (–N) for 5 days. The youngest fully expanded leaf (YFEL) and the oldest leaf from the main stem and the roots were harvested. Metabolite responses for these tissues are reported separately in rows and genotypes separately in columns. Metabolites were detected and authenticated using either an LC-QQQ-MS method or a UPLC-Q-MS method with known standards. Metabolites in gray could not be detected by either of these two methods. Skewed data was transformed with either the natural log, logit or the square root. ANOVA was performed with Bonferroni *post hoc* tests at a significance level of 0.05. The response to N starvation was calculated using log2 (treatment/reference) where the treatment was the mean of the biological replicates (*n* = 4–6) grown under –N conditions and the reference was the mean of the biological replicates (*n* = 4–6) grown under +N conditions. No significant differences in response of that metabolite for any tissue or genotype is indicated by n.s. Allantoin was not detected (ND) in the oldest leaf. Significant log2 responses are shaded according to the legend with red indicating increased accumulation of the metabolite in response to N starvation and blue indicating reduced metabolite levels in response to N starvation. Dashed lines indicate enzymatic reaction that may be catalyzed by more than one enzyme. More than one arrow is only shown if the enzyme activity directions differ between 2 or more enzymes. Dotted lines indicate multiple enzymatic steps that are not presented.

**FIGURE 9:**
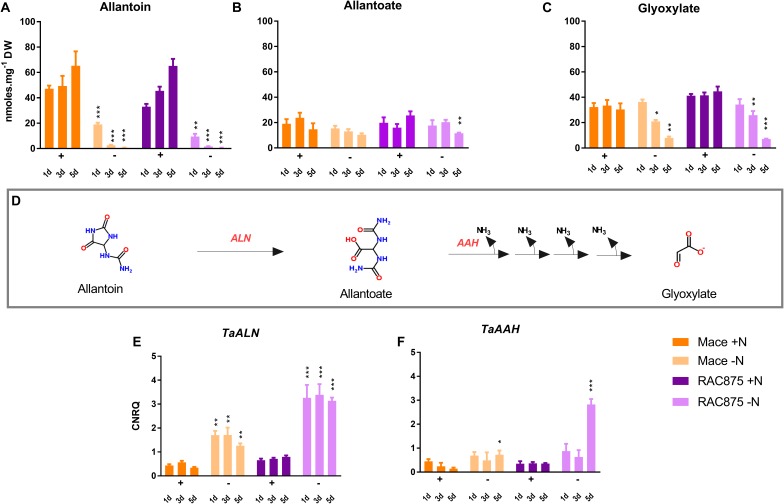
Purine metabolite levels and transcription of genes involved in allantoin catabolism **(D)** in the root in response to N starvation. Plants of the genotypes Mace and RAC875 were grown for 14 days with full nutrition before applying the treatments +N (1 mM N) or -N (0 mM N) for 1 day, 3 days or 5 days. *n* = 6 with bulking of 3 plants per n. Metabolites were extracted from root tissues for quantification of allantoin, allantoate and glyoxylate. Metabolites allantoin **(A)**, allantoate **(B)**, and glyoxylate **(C)** were separated and detected using LC-QQQ-MS. Peak area was normalized to the internal standard (norvaline) and divided by the dry weight of the sample. Metabolites were authenticated, and concentrations derived from regression analysis of standards. Transcriptional profile of the putative tonoplast localized equilibrative nucleoside allantoinase gene (*TaALN*, **E**) and the putative allantoate amidohydrolase (*TaAAH*, **F**) expressed as calibrated normalized relative quantities (CNRQ). Statistical significance between treatments within a single time point and a single genotype at ^∗∗∗^*p* ≤ 0.001, ^∗∗^*p* ≤ 0.02, ^∗^*p* ≤ 0.05 as determined by *T*-test analysis and *post hoc* analysis using the Holm–Sidak method.

To dissect changes occurring more widely in the purine metabolic pathway in response to N starvation, a quantitative metabolomics method (LC-QQQ-MS/MS) was optimized for detection of purine and pyrimidine nucleotides and catabolites based on a method by Guo et al. (2013). These results are presented in Figures 8, 9, 11.

The metabolomics data is presented as log2 response of plants grown under severe N stress (5 days -N) as compared to control (5 d +N) plants. The response of each metabolite extracted from individual tissues (YFEL, Oldest leaf and Root) in Mace and RAC875 is presented with blue indicating significant down-regulation and red indicating significant up-regulation (Figure 8). As presented previously (Figure 6), RNA yields were reduced in both the YFEL and the roots of Mace and RAC875 in response to the N starvation but levels were unchanged in the oldest leaf (*p* < 0.05). Ribonucleases cleave RNA releasing nucleotides (i.e., ATP and GTP) which can be hydrolysed to monophosphates (AMP or GMP) and free phosphate ions. Although ATP and AMP could not be detected in this method, GMP levels were reduced by 6- to 8-fold under N starvation but only in the roots suggesting either local use or reduced synthesis (Supplementary Table 5 and Figure 8, *p* < 0.05). The nucleobase guanine can be cleaved from the ribose sugar; here guanine levels were reduced in the YFEL of Mace and unexpectedly in all tissues of RAC875 (Figure 8, *p* < 0.05). The nucleoside adenosine and the nucleobase adenine were not significantly responsive to the treatment in either genotype or time point. However, there was some accumulation of the deaminated and hydrolysed form hypoxanthine in the oldest leaf of both genotypes. Mace roots also accumulated hypoxanthine, but a reduction was observed in the roots of RAC875 in response to N starvation (Figure 8, *p* < 0.05). Guanine can also be deaminated to xanthine and similar to GMP responses, xanthine levels were reduced in the roots of both genotypes (Figure 8, *p* < 0.05). The accumulation of xanthine, hypoxanthine and urate in the oldest leaf of N starved Mace plants (Figure 8, *p* < 0.05) may indicate translocation of one of these or an up-stream metabolite from the roots. The purine catabolites down-stream of urate show a similar tissue-specific response with accumulation of allantoate, glyoxylate and succinate in the oldest leaf but a reduction in allantoin, glyoxylate and succinate in the roots (Figure 8, *p* < 0.05). Amino acids relevant to glyoxylate metabolism were also assessed. All amino acids were down-regulated in response to N starvation with the exception of L-glutamate which accumulated in the oldest leaf (Figure 8, *P* < 0.05), and was otherwise unchanged in the other tissues. L-glutamate is central to amino acid metabolism but also an important feedback regulator of ammonia assimilation, which has been noted as the reason for no diurnal or NO_3_^-^ responsive changes to L-glutamate levels as reviewed by Stitt et al. (2002). L-glutamate is further a precursor for chlorophyll synthesis and its accumulation in the oldest leaf may be related to a reduction of chlorophyll synthesis in this tissue which is experiencing N starvation induced senescence.

### Root Catabolism of Purine Intermediates Correlate With Enhanced Transcription of TaALN and TaAAH Under N Starvation

Under N sufficient conditions, RAC875 and Mace roots accumulated 7-fold and 15-fold more allantoin than the YFEL, respectively, whilst allantoin could not be detected in the oldest leaf (Supplementary Table 5). Under severe N stress, allantoin and specific downstream catabolites were reduced in the roots with accumulation of allantoate and glyoxylate in the leaves, which could suggest translocation (Figure 8). To assess whether allantoin is degraded locally in the roots serving as an N source or whether it is exported to sink tissues, root allantoin catabolism was investigated in detail (Figure 9). Root accumulation of allantoin (Figure 9A) and catabolites allantoate (Figure 9B) and glyoxylate (Figure 9C) are presented along-side with transcriptional profiles of allantoinase (*TaALN*, Figure 9E) and allantoate amidohydrolase (*TaAAH*, Figure 9F). *TaALN* encodes a protein responsible for hydrolysis of allantoin to allantoate, and *TaAAH* encodes the enzyme to catabolise allantoate to ureidoglycine thereby releasing nitrogen in the form of ammonia (Figure 9D). Root allantoin levels were reduced early in both genotypes; reductions of 19- to 28- fold were observed for Mace and RAC875 plants grown under mid N stress. This correlated with a 3.9- to 5- fold increase in expression of *TaALN* in those plants grown under mild N stress (Figure 9, *p* < 0.05). Despite a reduction in allantoin levels, the catabolite allantoate was unchanged in the roots of Mace and reduced in RAC875 roots only when grown under severe N stress. *TaAAH* was transcriptionally induced by 5-fold in Mace and 8-fold in RAC875 roots when plants were grown under severe N stress (Figure 9, *p* < 0.05), which in concurrence with the allantoate results suggests that allantoate is catabolised in the roots of plants grown under severe N stress. The loss of one molecule of allantoin should lead to one additional molecule of allantoate as indicated in the chemical structures shown (Figure 9), and since *TaAAH* was only induced under severe N stress, the lack of change in allantoate under mild and mid- N stress suggests that either allantoin or allantoate is translocated to the shoots. Although uredioglycolate and the other intermediate catabolic metabolites could not be detected using this method, glyoxylate, the carbon product after release of all amine groups, was detected. Glyoxylate was reduced in the roots of plants grown under mid and severe N stress (Figures 8, 9, *p* < 0.05), however, glyoxylate levels were enhanced in response to severe N stress in the above-ground leaves (YFEL and oldest leaf, Figure 8, *p* < 0.05) further supporting a possible translocation from the roots.

### Xanthine and Allantoin Can Serve as N Sources When Supplied Externally to N Starved Wheat Plants

**FIGURE 10:**
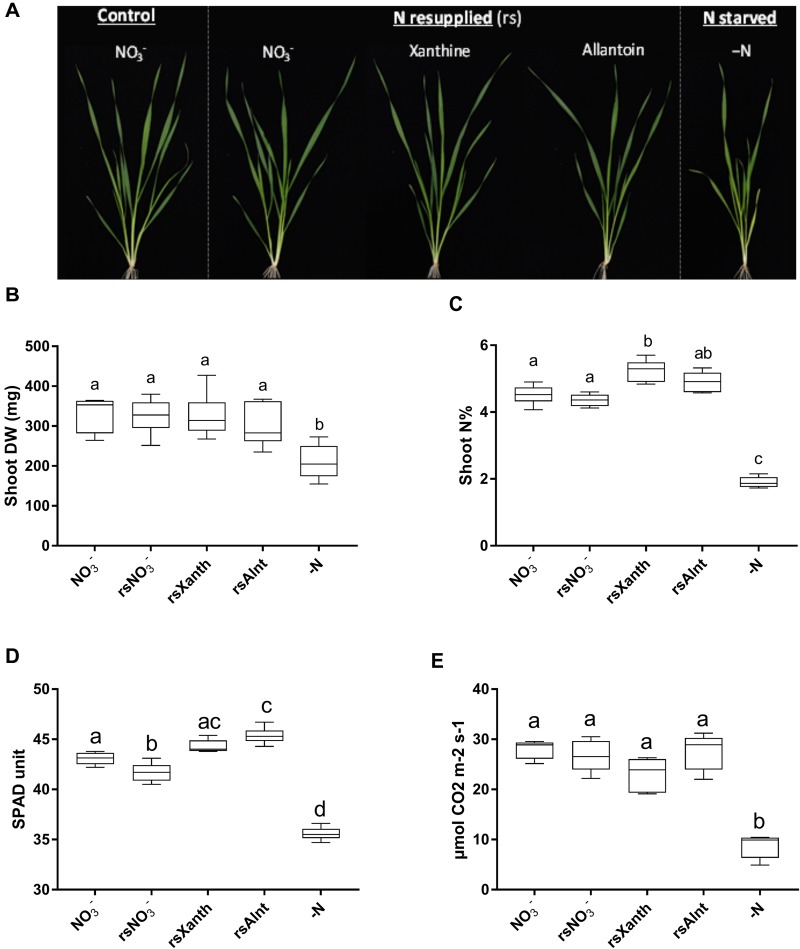
The effect of resupplying inorganic and organic N sources on the growth and physiology of N starved wheat plants. Mace seedlings were grown in a hydroponic system supplied with 1 mM NO_3_^-^ for 14 days and then starved of N for 24 h (*Experiment 3*). N was resupplied as 1 mM NO_3_^-^ (rsNO_3_^-^), 0.25 mM xanthine (rsXant), 0.25 mM allantoin (rsAlnt) or not (–N) for eight days; a subset of plants was kept at constant NO_3_^-^ supply throughout the experiment (NO_3_^-^). All photographs and measurements were taken after eight days of the treatment. **(A)** Photographs of the plants **(B)** shoot dry weight (DW), **(C)** shoot N concentration (%), **(D)** chlorophyll content expressed in SPAD unit and **(E)** photosynthetic rate. Letters indicate significant different between treatments per time point by one-way ANOVA with Tuckey’s correction (*p* < 0.05).

Purines and pyrimidines are commonly found in root exudates (Dennis et al., 2010) and they can also be taken up by plants when supplied exogenously (Desimone et al., 2002). However, how nucleotides are taken up from their external environment, and whether as a derivative or intact is not yet confirmed. Ureide Permeases (UPSs) responsible for xylem and phloem loading of allantoin and other purine derivatives has been identified in bean and Arabidopsis (Desimone et al., 2002; Pélissier et al., 2004; Schmidt et al., 2006; Lescano et al., 2016). To assess whether the N recycled from purine catabolism can be effectively used to support growth in wheat, Mace plants were grown hydroponically for 14 days with NO_3_^-^, starved for 24 h (mild N stress) then resupplied with either NO_3_^-^ (rsNO_3_^-^), xanthine (rsXanth) or allantoin (rsAlnt) as the sole source of N (Figure 10). Control plants were either grown with steady supply of NO_3_^-^ supply without a period of N starvation or maintained for 8 days under N starvation (-N). Visual observation of plants after resupply of NO_3_^-^, xanthine or allantoin did not reveal obvious differences to control plants grown under constant NO_3_^-^ supply. In contrast, yellowing of the older leaves from plants maintained without N for 8 days (-N) was evident (Figure 10A). The shoot dry weight (DW) was equivalent between plants resupplied with xanthine and allantoin as those resupplied with NO_3_^-^ or grown under constant NO_3_^-^, and all were greater than that measured in the -N plants (Figure 10B). The shoot N content (N%) was greater in plants resupplied with xanthine as compared to the -N control but was equivalent between allantoin resupply and the constant NO_3_^-^ control (Figure 10C). The photosynthetic rate of plants resupplied with xanthine and allantoin was also comparable to that of the NO_3_^-^ (constant and resupplied) plants, whilst plants deprived of N had significantly reduced photosynthetic rates (Figure 10E). Plants re-supplemented with xanthine and allantoin accumulated more chlorophyll than control plants growing on constant NO_3_^-^ whilst levels were lowest in the -N control (Figure 10D).

### Pyrimidine Nucleosides Accumulate in N Starved Wheat Plants

**FIGURE 11:**
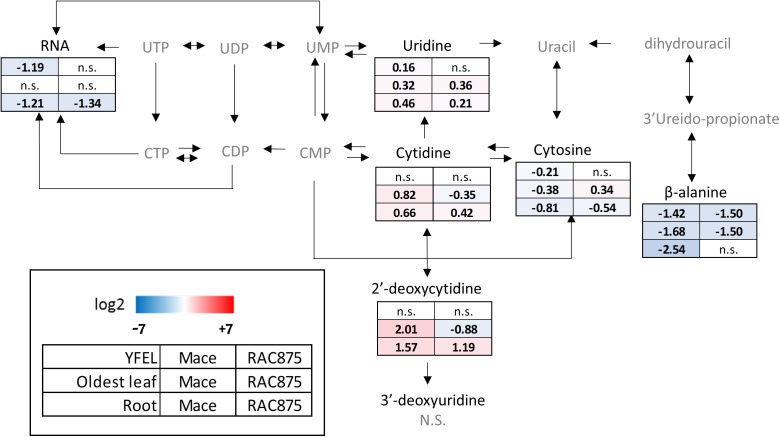
Metabolic map of pyrimidine metabolism in bread wheat in response to N starvation. Bread wheat genotypes Mace and RAC875 were grown for 14 days under N sufficient conditions (+N) before transfer to N starvation conditions (–N) for 5 days. The youngest fully expanded leaf (young leaf) and the oldest leaf from the main stem and the roots were harvested. Metabolite responses for these tissues are reported separately in rows and genotypes separately in columns. Metabolites were detected and authenticated using either an LC-QQQ-MS method or a UPLC-Q-MS method. Metabolites in gray could not be detected by either of these two methods. Skewed data was transformed with either the natural log, logit or the square root. ANOVA was performed with Bonferroni *post hoc* tests at a significance level of 0.05. The response to N starvation was calculated using log2(treatment/reference) where the treatment was the mean of the biological replicates (*n* = 4–6) grown under –N conditions and the reference was the mean of the biological replicates (*n* = 4–6) grown under +N conditions. No significant differences in response of that metabolite for any tissue or genotype is indicated by n.s. Significant log2 responses are shaded according to the legend with red indicating increased accumulation of the metabolite in response to N starvation and blue indicating reduced metabolite levels in response to N starvation. Dashed lines indicate enzymatic reaction that may be catalyzed by more than one enzyme. More than one arrow is only shown if the enzyme activity directions differ between 2 or more enzymes.

Tissues sourced from N starved wheat plants accumulated significantly higher levels of the purine nucleobase adenine and the pyrimidine nucleoside uridine than the other detectable nucleosides/bases (Supplementary Table 5). This could be related to the role of adenine and uridine as cofactors in metabolism and for the synthesis of nucleotide sugars such as ADP-glucose and UDP-glucose (Zrenner et al., 2006). The pyrimidine nucleosides uridine and cytidine derive from the nucleotides UTP and CTP respectively; these nucleotides could not be detected using this method. Uridine was accumulated in all tissues of both genotypes in response to severe N stress (Figure 11, *p* < 0.05) except for the youngest leaf of RAC875 where RNA yields were also unchanged (Figure 11). Absolute uridine levels were significantly higher than cytidine in all tissues and genotypes, for example, uridine levels were detected at more than 1000-fold that of cytidine levels measured in the oldest leaf of N starved Mace plants (Supplementary Table 5).

Cytidine and 2′-deoxycytidine also accumulated in the roots of both genotypes and the oldest leaf of Mace, however, levels were reduced in the oldest leaf of RAC875 (Figure 11, *p* < 0.05). The opposite response to that of cytidine was seen with the nucleoside cytosine with a reduction in the roots of both genotypes and the oldest leaf of Mace but an accumulation in the oldest leaf of RAC875 (Figure 11, *p* < 0.05). β-alanine levels were decreased in all tissues of both genotypes in response to N starvation (Figure 11, *p* < 0.05) except for no response in the roots of RAC875 (Figure 11). 3′-deoxyuridine is the precursor to thymidine, a component of DNA, and levels were unchanged in response to N starvation (Figure 11).

## Discussion

Recent evidence suggests that under nutrient stress conditions, novel pathways of ribosome and rRNA turn-over exist independent of the constitutive quality control mechanisms (Bassham and MacIntosh, 2017). Although characterization of this selective autophagy pathway is still in its infancy, there was evidence in the literature suggesting that wheat has a large pool of free nucleotides and nucleosides prompting this investigation into the nutritional benefits of nucleotide catabolism. Early tillering wheat plants starved of N for 5 days resulted in a 50% reduction of total N and an approximate 19% reduction in shoot NO_3_^-^. The N starved plants were thereby experiencing severe N deficiencies leading to visible signs of chlorosis of the older leaves. A comparison of shifts in the shoot N pool demonstrated that across genotypes NO_3_^-^ dominated the N pool (15% of the total N pool as compared to nucleic acids which represented only 3.5–4.4% of the total N pool) in plants grown under N sufficient conditions (Figure 2). However, due to the greater depletion of NO_3_^-^ after 5 days of N starvation, the N from nucleic acids (3.6–4.8% of the total N pool) was more comparable with that from NO_3_^-^ (5.2–6% of the total N pool), (Figure 2).

### An N Efficient Wheat Genotype Responds to Mild N Stress

Genotypes contrasting in NUE were carefully selected for this study as little is known about how they store, utilize and respond to N limitations when they are establishing their tillers. Although the shoot fresh weight of RAC875 was greater than Mace, the total shoot nitrogen content was equivalent between Mace and RAC875 (Supplementary Table 2) suggesting that RAC875 is more responsive to N supply in this system. However, under N starvation conditions, both genotypes restricted growth and had comparable shoot N content; restricting growth is an important adaption to the environment ensuring that there is an adequate supply of N during flowering and grain filling. However, we cannot presume that the restriction at early vegetative stages observed here continues into the reproductive stages of development.

This system enabled us to dissect differences in the accumulation and distribution of nitrate, an important form of inorganic nitrogen. The uptake, translocation, storage and assimilation of NO_3_^-^ is critical to later support the emerging flag leaf. For example, the YFEL of Mace accumulated 18% higher levels of NO_3_^-^ than RAC875 under N sufficient conditions (Figure 5). This perhaps indicates differences in storage of NO_3_^-^; the highest proportion of cellular NO_3_^-^ is likely to be in the vacuole as was demonstrated in barley (Zhen et al., 1991). However, on a whole shoot basis, NO_3_^-^ levels were comparable between Mace and RAC875 (Supplementary Table 2). Although RNA yields in whole shoots were not significantly different between genotypes (Figure 3B), Mace roots responded to the N stress earlier than RAC875, leading to depletion of RNA under mild N starvation (Figure 6). Absolute DNA and RNA yields were reduced in RAC875 shoots under N starvation whilst only RNA levels were reduced in Mace shoots (Figure 3B). However, since thymidine was not detected in our quantitative metabolomics screen, it was impossible to differentiate between nucleotide degradation products derived from the catabolism of RNA from those derived from the catabolism of DNA.

### RNA Catabolism Occurs in N Starved Wheat Roots

Since RNA yields were reduced in the shoots of both genotypes, we questioned whether RNA catabolism could differ between tissues to redistribute nutrient resources. N stress caused inhibition of the YFEL and oldest leaf but no changes to root growth (Figure 4); we have similarly demonstrated root proliferation in wheat plants grown in N deficient soils (Melino et al., 2015). Under N sufficient conditions, the YFEL accumulated more RNA than the roots or the oldest leaf as expected from a highly metabolically active tissue (Figure 6). RNA yields were reduced in Mace roots from mild N starvation (Figure 6). Reduced RNA yields in the roots correlated with induction of *TaRNS2, TaENT1* and *TaENT3* (Figure 7C). Metabolic analysis further supported that nucleotide catabolism was up-regulated in N starved roots; with reduced levels of GMP, guanine, xanthine, allantoin, allantoate, glyoxylate and succinate under severe N stress (Figure 8). Results from a comparison of root accumulation of three purine metabolites (allantoin, allantoate and glyoxylate) with the transcriptional response of genes encoding allantoin catabolism (*TaALN, TaAAH*) suggests that whilst allantoin may be catabolised through to glyoxylate in the roots of severe N starved plants, allantoin or allantoate is more likely to be translocated to above-ground tissue under mild N stress (Figure 9). This was supported by our results showing that allantoate accumulated in the oldest leaf whilst glyoxylate accumulated in both the YFEL and the oldest leaf (Figure 8).

### Young Wheat Leaves Shift From Local RNA Degradation to Importing Nucleosides

RNA yields were reduced in the YFEL of Mace plants grown under severe N stress. A lack of induction of *TaRNS2* suggests that vacuolar RNA catabolism did not occur in this tissue so that reduced RNA levels are likely due to reduced synthesis. The significant induction of *TaENT3* in the YFEL of N starved Mace plants (Figure 7A) may indicate reliance on imported nucleosides rather than local degradation of RNA. There was like-wise no treatment effect on RNA yields in RAC875 plants supported by constitutive *TaRNS2* expression (Figure 7), however, reduced RNA yields over the 5 days of development was observed in the YFEL of RAC875 (Figure 6), perhaps indicating reduced RNA synthesis in this tissue. One must also consider that these plants had two additional tillers with younger leaves which may demand reallocation of the available N.

### Older Wheat Leaves Accumulate Nucleosides Despite an Apparent Reduction in RNA Synthesis

RNA abundance was more than 10-fold lower in the oldest leaf than that in the YFEL and there was no significant treatment effect (Figure 6). Despite reduced RNA yields, the oldest leaf accumulated a large pool of free nucleosides and derivatives, which were enriched under N starvation (Supplementary Table 5). This was particularly evident for pyrimidines where under N starvation the oldest leaf accumulated 9-fold more pyrimidine nucleosides than the root and 1.7-fold more than the YFEL when averaged across both genotypes (Supplementary Table 5). It is possible that RNA catabolism occurred developmentally earlier than was measured in this study and that the large pool of free nucleoside derivatives is the product of that process.

RNA catabolism was also not observed in the oldest leaf of N starved Mace and RAC875 plants (Figure 6) and unexpectedly transcription of *TaRNS2* and *TaENT1* was enhanced (Figure 7B). It was previously shown that *TaRNS2* was induced in leaves at the transcriptional level under senescence and Pi starvation (Taylor et al., 1993) and furthermore, RNS2 knock-down mutants were more sensitive to Pi starvation than the wild type controls (Bariola et al., 1999). We propose that transcription of *RNS2, ENT1* and *ENT3* is regulated by a signal of nutritional stress rather than by vacuolar accumulation of RNA or nucleosides.

### N-Rich Purine Nucleosides Support Plant Growth

A comparison of root specific accumulation of purine and pyrimidine metabolites in N starved wheat plants showed a general trend of degradation of purine nucleoside catabolites (Figure 8) but an accumulation of pyrimidine nucleosides (Figure 11). This lends support to our hypothesis that the N-rich purine nucleosides, both free nucleosides derived from salvage pathways or from import and those derived from RNA catabolism (Figure 1), can support the N requirements of growth. To evaluate this further, N starved wheat plants were exogenously supplied with xanthine or allantoin and their growth was monitored for 8 days. The results demonstrated that N starved wheat plants resupplied with these purine metabolites, could maintain photosynthetic rate and shoot dry weight comparably to the NO_3_^-^ controls (Figure 10). In fact, the resupply of xanthine or allantoin resulted in higher chlorophyll content. In contrast to our findings in wheat, 11 days old Arabidopsis seedlings were better able to utilize ammonium NO_3_^-^ than allantoin or xanthine as the sole source of nitrogen (Desimone et al., 2002). This may be due to the preferential and rapid uptake and assimilation of ammonium by Arabidopsis rather than NO_3_^-^. Alternatively, it could be due to differences in purine uptake between plant species or different methodologies applied [steady-state supply by Desimone et al. (2002) as compared to N starvation and resupply here].

The N starved wheat plants resupplied with xanthine had a greater shoot %N than those either resupplied with NO_3_^-^ or grown under constant NO_3_^-^ supply without any period of N starvation. Since equivalent molecules of nitrogen were supplied in the form of xanthine and NO_3_^-^, this must indicate that the xanthine derived N was assimilated. Another point to consider is that plants which were resupplied with xanthine and allantoin were provided with four molecules of carbon that were not present with the resupply of NO_3_^-^ and may have some impact on the recovery of these plants. None-the-less, this experiment demonstrated that purines can be taken up by roots and deaminated to release and recycle the nitrogen back into primary metabolism. Allantoin and xanthine can be taken up into roots and then translocated to the shoot by the activity of proteins in the ureide permease family; evidenced by the fact that UPS5 defective mutants hyperaccumulate allantoin in their roots and have reduced shoot allantoin levels (Desimone et al., 2002; Lescano et al., 2016). Translocation of ^15^N labeled allantoin or allantoate would need to be experimentally verified in wheat, however, allantoin is known to be translocated in Arabidopsis and bean by the activity of ureide permease (UPS) transporters (Desimone et al., 2002; Pélissier et al., 2004; Schmidt et al., 2004, 2006; Lescano et al., 2016), which are yet to be identified in wheat. Curci et al. (2017) demonstrated, using an RNA-seq approach, that a ureide permease (based on sequence identity to *AtUPS5* which transports allantoin, Schmidt et al., 2006) was down-regulated in durum wheat leaf/stem at reproductive stages of development in response to N deficiency. However, a putative nucleobase symporter and a probable purine permease were up-regulated in the roots in response to N limitation. An examination of UPS activity in wheat is needed to better understand how purines from the root support reproductive above-ground growth.

## Conclusion

On the premise that wheat stores high levels of free nucleotides (Sawert et al., 1988), we investigated the contribution of nucleic acid catabolism to N recycling and the support of early vegetative growth and development. A comparison of shoot N pools revealed that nucleic acids could represent a vital source of N (∼4% of the N pool) for plants grown under N stress when shoot nitrate levels are low. Supplying purine derivatives exogenously was shown to support the recovery of N starved wheat plants as efficiently as exogenous nitrate supply. Furthermore, RNA and purine catabolism were induced in rapidly growing roots of plants exposed to N starvation, as were transcriptional levels of genes encoding RNA degradation and nucleoside transport. On the other-hand, total pyrimidine nucleotides and derivatives accumulated 2- to 6-fold in N starved plants and are therefore unlikely contributors of N in the tissues examined. These results collectively support our hypothesis that N recovered from purine catabolism enters primary metabolism to maintain plant growth and cellular processes under N limiting conditions.

## Author Contributions

SH and MO initiated the project idea. VM designed and conducted the experiments, and wrote and edited the manuscript. AC designed, conducted, and analyzed an experiment. JG assisted with method development and conducted the experiments. TR developed the LC-MS method and analyzed all samples. UR, MO, and SH contributed to the design of the experiments and edited the manuscript.

## Conflict of Interest Statement

The authors declare that the research was conducted in the absence of any commercial or financial relationships that could be construed as a potential conflict of interest.

## Footnotes

1 imagej.nih.gov/ij/

2 https://www.ncbi.nlm.nih.gov/protein/

3 https://phytozome.jgi.doe.gov

4 http://pre.plants.ensembl.org/Triticum_aestivum/Info/Index

5 http://stableisotopefacility.ucdavis.edu/
