# Supplementary material for: RNA Catabolites Contribute to the Nitrogen Pool and Support Growth Recovery of Wheat
Source: Front Plant Sci. 2018 Nov 2;9:1539. doi: 10.3389/fpls.2018.01539 (PMC6230992; doi:10.3389/fpls.2018.01539)

Supplementary Material

**RNA CATABOLITES CONTRIBUTE TO THE NITROGEN POOL AND SUPPORT GROWTH RECOVERY OF WHEAT**

**Melino, V.J., Casartelli, A., George, J., Rupasinghe, T., Roessner, U., Okamoto, M. and Heuer, S*.**

*****Corresponding Author: sigrid.heuer@rothamsted.ac.uk

**Supplementary Table 1. Primer sequences and amplicon length for target bread wheat genes**

| Gene name | Forward primer sequence (5’-3’) | Reverse primer sequence (5’-3’) | Amplicon length (bp) |
| --- | --- | --- | --- |
| *TaADK* | CAACTGCCTCTGGCTTCTGG | GTCCTCGATGCTCTTCCCTTG | 207 |
| *TaENT1* | TACATGCAGGCTGTCGTCGC | TACAGGATTGCGCTTTGCC | 122 |
| *TaENT3* | TACAAAGGCAGCTTTCGAGA | CGGTAGTACTTCACGATGGG | 143 |
| *TaRNS2* | TATCCTGGTCTCCAACGGTA | GGACAATCTCCAGTGTCGAA | 268 |
| *TaCyc* | CAAGCCGCTGCACTACAAGG | AGGGGACGGTGCAGATGAA | 227 |
| *TaEFA* | CAGATTGGCAACGGCTACG | CGGACAGCAAAACGACCAAG | 227 |

**Supplementary Table 2. Shoot fresh weight, total nitrogen (N) and N metabolites measured in experiment 1**

**Supplementary Table 3. Identification of target bread wheat genes and orthologs**

| GOI | Arabidopsis# | *Brachypodium distachyon* (brachypodium) | Oryza sativa (rice)* | Triticum aestivum (bread wheat)** | References for orthologs |
| --- | --- | --- | --- | --- | --- |
| Adenosine kinas (*ADK*) | AT3g09820 (ADK1)  AT5g03300 (ADK2) | Bradi3g49230 | LOC_Os02g41590 | TGACv1_scaffold_473321_6AL  TGACv1_scaffold_499360_6BL  TGACv1_scaffold_526952_6DL | (Moffatt et al., 2000) |
| Equilibrative nucleoside transporter (*ENT1*) | At1g70330 | Bradi3g17700 | LOC_Os08g10450 | TGACv1_scaffold_472259_6AL  TGACv1_scaffold_502717_6BL  TGACv1_scaffold_526370_6DL | (Li and Wang, 2000;Hirose et al., 2005) |
| Equilibrative nucleoside transporter (*ENT3*) | At4g05120 | Bradi1g24960 | LOC_Os07g37100 | TGACv1_scaffold_16965_2AS  TGACv1_scaffold_3010_2BS  TGACv1_scaffold_15731_2DS | (Hirose et al., 2005) |
| Ribonuclease T2 (*RNS2*) | At2g39780 | Bradi2g57608 | LOC_Os01g67180 | TGACv1_scaffold_196569_3AL  TGACv1_scaffold_224141_3B  TGACv1_scaffold_252500_3DL | (Taylor et al., 1993) |

#NCBI; *Phytozome; **sequences identified in the present study

**Supplementary Table 4. Amino acid identity (% and grey shading) of the target bread wheat genes compared with the orthologous genes**

| **A** |  |  |  |  |  |  |
| --- | --- | --- | --- | --- | --- | --- |
|  | **TaRNS2_3AL** | **TaRNS2_3B** | **TaRNS2_3DL** | **BdRNS2** | **OsRNS2** | **AtRNS2** |
| **TaRNS2_3AL** |  | 97.8 | 96.1 | 78.1 | 72.9 | 44.7 |
| **TaRNS2_3B** | 97.8 |  | 95.3 | 78.8 | 73.9 | 45.1 |
| **TaRNS2_3DL** | 96.1 | 95.3 |  | 77.7 | 71.8 | 44.3 |
| **BdRNS2** | 78.1 | 78.8 | 77.7 |  | 74.5 | 47.7 |
| **OsRNS2** | 72.9 | 73.9 | 71.8 | 74.5 |  | 44.8 |
| **AtRNS2** | 44.7 | 45.1 | 44.3 | 47.7 | 44.8 |  |

| **B** |  |  |  |  |  |  |
| --- | --- | --- | --- | --- | --- | --- |
|  | **TaENT1_6AL** | **TaENT1_6BL** | **TaENT1_6DL** | **BdENT1** | **OsENT1** | **AtENT1** |
| **TaENT1_6AL** |  | 99.0 | 98.8 | 92.2 | 81.6 | 62.6 |
| **TaENT1_6BL** | 99.0 |  | 98.8 | 92.6 | 82.3 | 63.1 |
| **TaENT1_6DL** | 98.8 | 98.8 |  | 92.4 | 82.3 | 62.6 |
| **BdENT1** | 92.2 | 92.6 | 92.4 |  | 84.2 | 62.3 |
| **OsENT1** | 81.6 | 82.3 | 82.3 | 84.2 |  | 63.3 |
| **AtENT1** | 62.6 | 63.1 | 62.6 | 62.3 | 63.3 |  |

| **C** |  |  |  |  |  |  |
| --- | --- | --- | --- | --- | --- | --- |
|  | **TaENT3_2AS** | **TaENT3_2BS** | **TaENT_2DS** | **BdENT3** | **OsENT3** | **AtENT3** |
| **TaENT3_2AS** |  | 99.5 | 99.5 | 88.8 | 82.1 | 64.2 |
| **TaENT3_2BS** | 99.5 |  | 100.0 | 89.0 | 81.9 | 64.2 |
| **TaENT_2DS** | 99.5 | 100.0 |  | 89.0 | 81.9 | 64.2 |
| **BdENT3** | 88.8 | 89.0 | 89.0 |  | 83.5 | 64.2 |
| **OsENT3** | 82.1 | 81.9 | 81.9 | 83.5 |  | 65.9 |
| **AtENT3** | 64.2 | 64.2 | 64.2 | 64.2 | 65.9 |  |

| **D** |  |  |  |  |  |  |  |
| --- | --- | --- | --- | --- | --- | --- | --- |
|  | **TaADK_6AL** | **TaADK_6BL** | **TaADK_6DL** | **BdADK** | **OsADK** | **AtADK1** | **AtADK2** |
| **TaADK_6AL** |  | 98.3 | 99.1 | 94.8 | 93.0 | 82.8 | 82.9 |
| **TaADK_6BL** | 98.3 |  | 98.8 | 95.4 | 93.3 | 82.9 | 83.2 |
| **TaADK_6DL** | 99.1 | 98.8 |  | 95.1 | 93.3 | 83.1 | 83.2 |
| **BdADK** | 94.8 | 95.4 | 95.1 |  | 94.5 | 82.6 | 83.5 |
| **OsADK** | 93.0 | 93.3 | 93.3 | 94.5 |  | 81.1 | 82.0 |
| **AtADK1** | 82.8 | 82.9 | 83.1 | 82.6 | 81.1 |  | 92.5 |
| **AtADK2** | 82.9 | 83.2 | 83.2 | 83.5 | 82.0 | 92.5 |  |

**Supplementary Figure 1.** **The effect of N starvation on tissue accumulation of ammonium and total basic amino acid.** Ammonium and 20 standard free amino acids were identified by HPLC and authenticated with standards of known concentrations. Data was normalised to an internal standard (norvaline) and to the sample dry weight (DW) and expressed as per 1 g DW^-1^. Ammonium and the sum of 20 standard amino acids is presented.


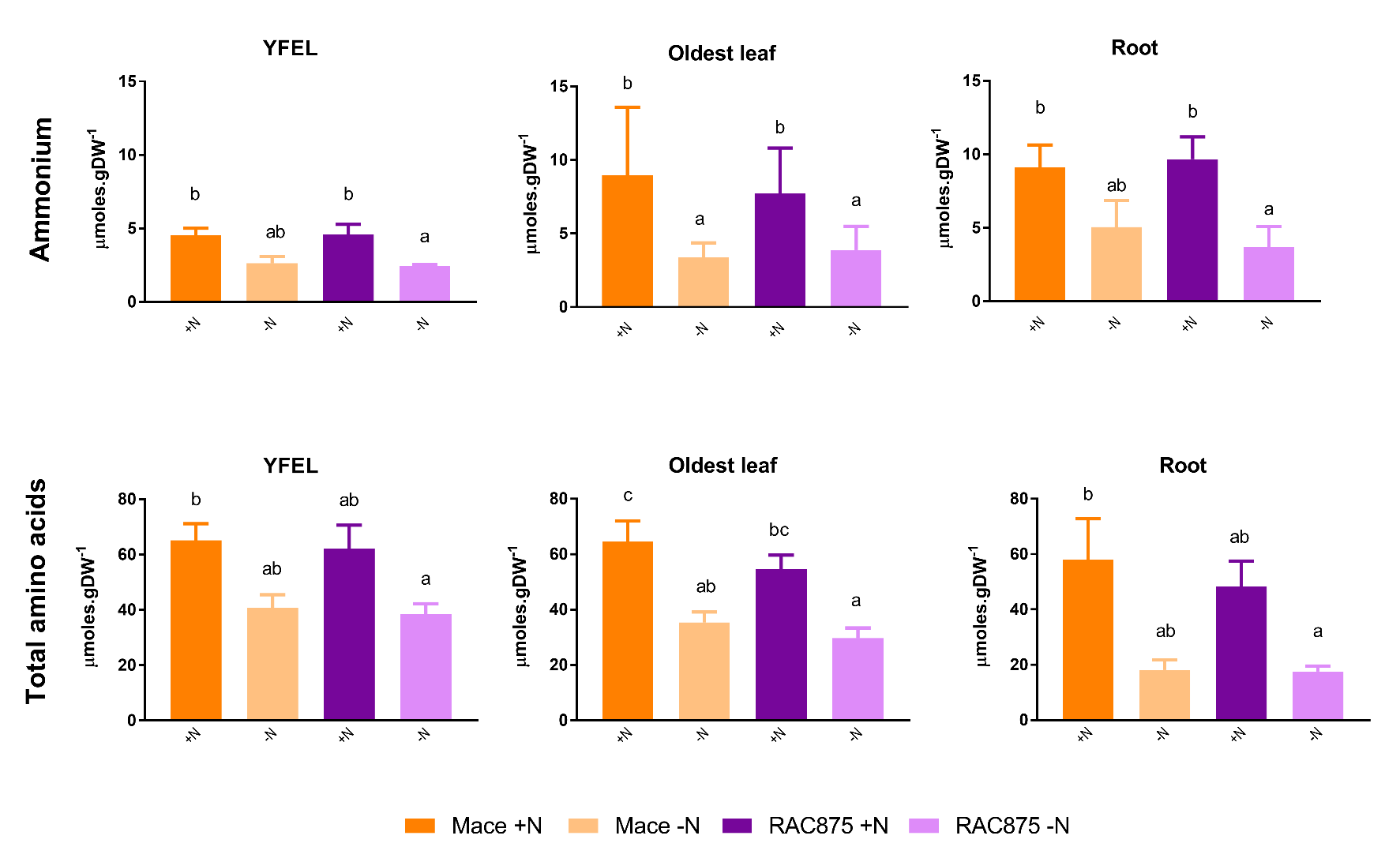

Supplement: Supplementary file 2 [file Table_1.docx]
